# Spray drying OZ439 nanoparticles to form stable, water-dispersible powders for oral malaria therapy

**DOI:** 10.1186/s12967-019-1849-8

**Published:** 2019-03-22

**Authors:** Kurt D. Ristroph, Jie Feng, Simon A. McManus, Yingyue Zhang, Kai Gong, Hanu Ramachandruni, Claire E. White, Robert K. Prud’homme

**Affiliations:** 1 Department of Chemical and Biological Engineering, Princeton University, Princeton, NJ 08854 USA; 2 Department of Civil and Environmental Engineering, Princeton University, Princeton, NJ 08854 USA; 3 Andlinger Center for Energy and the Environment, Princeton University, Princeton, NJ 08854 USA; 4 Medicines for Malaria Venture, Route de Pré-Bois 20, 1215 Meyrin, Switzerland

**Keywords:** Nanocarrier, Hydrophobic ion pairing, Malaria, Spray drying, Drug delivery, Oral therapeutic, Drug solubilization, OZ439, Artefenomel, Flash NanoPrecipitation

## Abstract

**Background:**

OZ439 is a new chemical entity which is active against drug-resistant malaria and shows potential as a single-dose cure. However, development of an oral formulation with desired exposure has proved problematic, as OZ439 is poorly soluble (BCS Class II drug). In order to be feasible for low and middle income countries (LMICs), any process to create or formulate such a therapeutic must be inexpensive at scale, and the resulting formulation must survive without refrigeration even in hot, humid climates. We here demonstrate the scalability and stability of a nanoparticle (NP) formulation of OZ439. Previously, we applied a combination of hydrophobic ion pairing and Flash NanoPrecipitation (FNP) to formulate OZ439 NPs 150 nm in diameter using the inexpensive stabilizer hydroxypropyl methylcellulose acetate succinate (HPMCAS). Lyophilization was used to process the NPs into a dry form, and the powder’s in vitro solubilization was over tenfold higher than unprocessed OZ439.

**Methods:**

In this study, we optimize our previous formulation using a large-scale multi-inlet vortex mixer (MIVM). Spray drying is a more scalable and less expensive operation than lyophilization and is, therefore, optimized to produce dry powders. The spray dried powders are then subjected to a series of accelerated aging stability trials at high temperature and humidity conditions.

**Results:**

The spray dried OZ439 powder’s dissolution kinetics are superior to those of lyophilized NPs. The powder’s OZ439 solubilization profile remains constant after 1 month in uncapped vials in an oven at 50 °C and 75% RH, and for 6 months in capped vials at 40 °C and 75% RH. In fasted-state intestinal fluid, spray dried NPs achieved 80–85% OZ439 dissolution, to a concentration of 430 µg/mL, within 3 h. In fed-state intestinal fluid, 95–100% OZ439 dissolution is achieved within 1 h, to a concentration of 535 µg/mL. X-ray powder diffraction and differential scanning calorimetry profiles similarly remain constant over these periods.

**Conclusions:**

The combined nanofabrication and drying process described herein, which utilizes two continuous unit operations that can be operated at scale, is an important step toward an industrially-relevant method of formulating the antimalarial OZ439 into a single-dose oral form with good stability against humidity and temperature.

**Electronic supplementary material:**

The online version of this article (10.1186/s12967-019-1849-8) contains supplementary material, which is available to authorized users.

## Background

Great strides have been taken in the fight to eradicate malaria, and the number of deaths from the disease has been reduced by as much as 62% over the past decade and a half [1]. However, malaria remains one of the most prevalent infectious diseases in the world, infecting 219 million individuals and killing 435,000 in 2017 [2]. Among the most successful tools in this fight is the artemisinin combination therapy (ACT) [3], but recent years have seen the development of resistance to ACT therapy [4]. Resistance is attributed, in part, to poor patient adherence to the ACT regimen [5], which consists of twelve pills taken over the course of 3 days [5, 6]. A single-dose malaria cure—ideally, in oral dosage form—is therefore highly desirable.

OZ439 is a promising antimalarial drug that is being pursued as a single-dose oral malaria therapeutic, in part because of its high potency and the fact that resistance to it has not been observed [7–10]. To formulate as a single dose, the bioavailability of OZ439 needs to be increased. This work is a continuation of our previous study, in which we formulated OZ439 into polymeric nanoparticles via the scalable nanofabrication process Flash NanoPrecipitation (FNP) using Hypromellose Acetate Succinate as a stabilizer [11]. Formulation into NPs helps OZ439 overcome its poor oral bioavailability via two mechanisms: first, the high surface-to-volume ratio of a NP formulation increases dissolution rate; and second, x-ray powder diffraction (XRPD) and differential scanning calorimetry (DSC) profiles showed that OZ439 within the NPs is amorphous, rather than crystalline, leading to higher solubility and faster dissolution kinetics [11].

In this paper we focus on the translation of the earlier laboratory study to a large-scale process that could be used in a commercial, cost effective, good manufacturing practice (GMP) drug production line. The key elements of this translation are (1) moving the NP formation process from the Confined Impinging Jet (CIJ) mixer to the large-scale and continuous Multi-Inlet Vortex Mixer (MIVM), and (2) moving from lyophilization to continuous spray drying to produce dry powders. The characterization of NP stability and crystallinity are compared for samples made by the CIJ versus the MIVM process. Spray drying conditions including inlet temperature and gas flow rate are optimized. The dissolution kinetics of the powders in simulated gastric fluid and intestinal fluids in fasted and fed state conditions are presented. Results from a 6-month aging study show that the spray dried NPs are completely stable over this time period. An interesting final conclusion is that the dissolution kinetics of OZ439 NP powders processed by spray drying are superior to those of lyophilized NP powders.

## Materials and methods

### Materials

Affinisol HPMCAS 126 G (> 94% purity) and Methocel E3 Premium LV Hydroxypropyl Methylcellulose (HPMC E3) were generously provided by Dow Chemical. Tetrahydrofuran (HPLC grade, 99.9%), methanol (HPLC grade, 99.9% purity) and acetonitrile (HPLC grade, 99.9% purity) were purchased from Fisher Chemicals. Sodium oleate (> 97% purity) was purchased from TCI America. Fasted-state simulated intestinal fluid (FaSSIF), fed-state simulated intestinal fluid (FeSSIF) and fasted-state simulated gastric fluid (FaSSGF) powders were purchased from biorelevant.com. OZ439 mesylate was supplied by Medicines for Malaria Venture (MMV).

### Nanoparticle formation and characterization

Nanoparticles stabilized by HPMCAS and containing OZ439:oleate were formed via FNP. The FNP process has been described in detail previously [12, 13]. It involves two components: (1) rapid micromixing between a water-miscible organic solvent stream and an aqueous anti-solvent stream, and (2) kinetically arrested aggregation of the drug nanoparticle by adsorption of the stabilizer on its surface. The drug and stabilizing polymer are dissolved in the solvent stream. Upon mixing, which occurs on time scales of *O*(1) ms, the drug and amphiphilic portions of the stabilizing polymer adsorb on the growing aggregate and arrest growth. Nanoparticles from 25 to 450 nm can be produced with narrow size distributions and at high loadings.

**Fig. 1:**
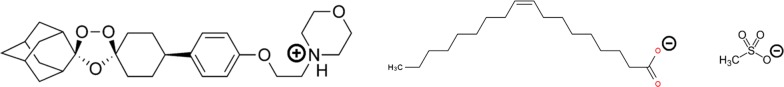
From left: OZ439 cation; oleate anion; mesylate anion

OZ439 is a synthetic trioxolane which was provided in a mesylate salt form (Fig. 1). In the mesylate salt form or free base form, the solubility of OZ439 is too high to create stable nanoparticles by antisolvent precipitation. When either of these forms is used, NPs initially formed during FNP rapidly succumb to Ostwald ripening and grow in size [14, 15]. To form stable NPs, sodium oleate was included in the organic feed stream and acted as a hydrophobic ion pairing agent. Cationic OZ439 and anionic oleate ions paired together, and the resulting complex was sufficiently hydrophobic to precipitate during the mixing step.

**Fig. 2:**
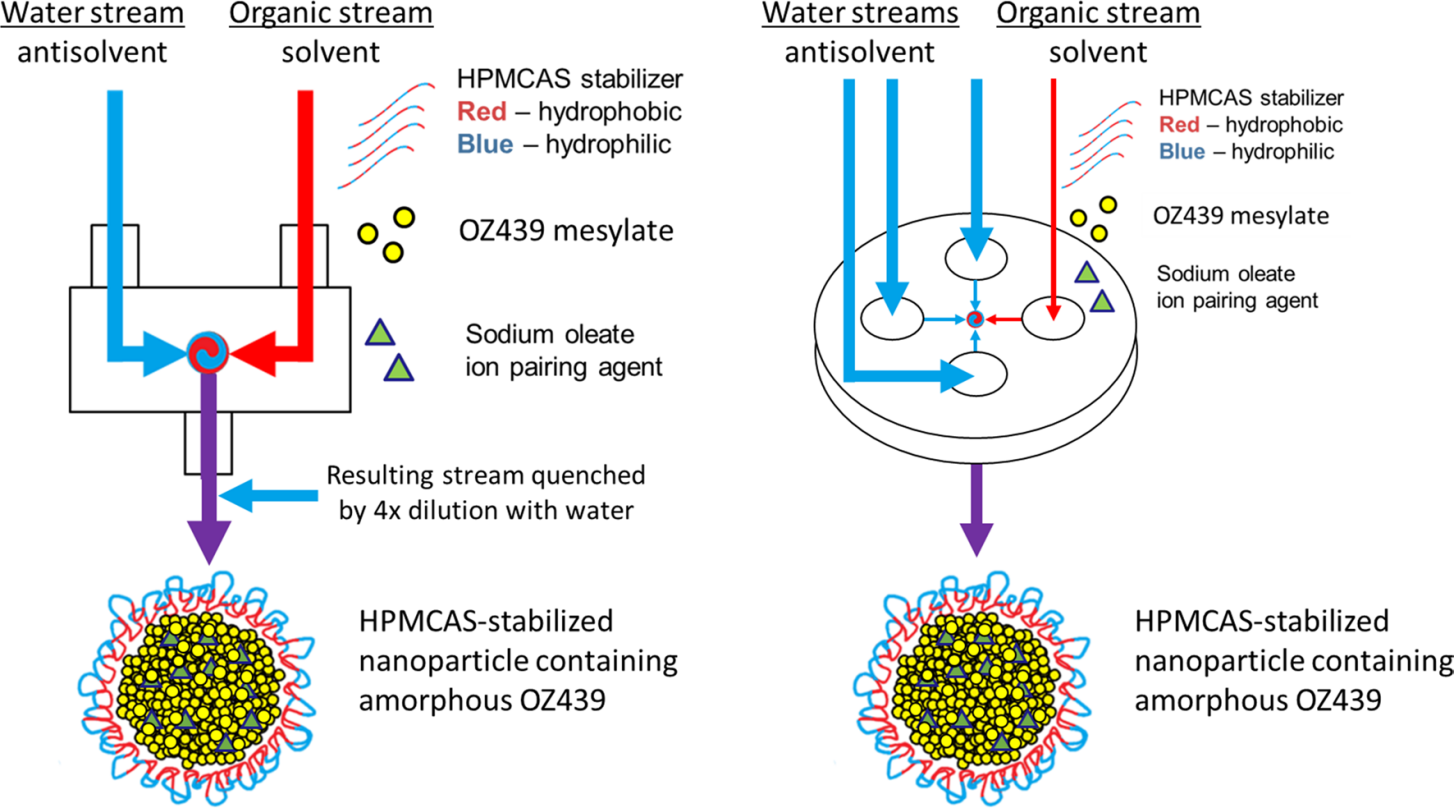
Schematic of CIJ mixer (left) and MIVM (right) to form OZ439 nanoparticles by FNP. The MIVM operates continuously and does not require the additional quenching step required of the CIJ mixing geometry

Previously, we had applied FNP to OZ439 using a two-inlet lab-scale CIJ mixer [11], which requires a quenching step to stabilize the NPs against Ostwald ripening. As the process is intended to be continuous and at large scale, we here employed a multi-inlet vortex mixer (MIVM) for the formation of nanoparticles. The MIVM allows unequal volumetric flow rates between its four inlets. By introducing three water antisolvent streams, each at three times the volumetric flow rate of the sole organic stream, the MIVM achieved the same final nanoparticle quenching by dilution of the organic solvent concentration, and thus bypassed the quenching step. Figure 2 is a schematic of the two mixers as applied to this process.

Nanoparticles were produced via FNP in the MIVM using sodium oleate as a hydrophobic counterion. OZ439 mesylate (5 mg/mL), sodium oleate (5.38 mg/mL), and HPMCAS 126 (5 mg/mL) were dissolved in a mixture of 33% methanol and 67% THF. This stream was loaded into a syringe and attached to the MIVM, along with three syringes containing DI water. Using a syringe pump (Harvard Apparatus, Massachusetts, USA), the organic stream and water streams were fed into the MIVM at controlled flow rates. The organic stream was fed at 16 mL/min, and each of the water streams was fed at 48 mL/min, such that the resulting NP suspension contained 10% organic solvent by volume.

Nanoparticle mean size, size distribution, and polydispersity were measured by dynamic light scattering (DLS) in a Malvern Zetasizer Nano (Malvern Instruments, Worcestershire, United Kingdom). Following formation, nanoparticle samples were diluted tenfold in DI water immediately prior to measurement to reduce multiple scattering. The Zetasizer was operated at room temperature and used a detection angle of 173°. Measurements were taken in triplicate. DLS data were processed with Malvern’s software using a distribution analysis based on a cumulant model. The cumulant analysis is defined in International Organization for Standardization (ISO) standard document 13321. The calculations of PDI are defined in the ISO standard document 13321:1996 E.

### Lyophilization conditions

In order to process nanoparticle suspensions into dry powders for long-term storage and ease of shipping, a drying unit operation like lyophilization or spray drying was required. In lyophilization, a frozen sample is subjected to low temperatures and pressures, and ice and frozen organic solvents are removed by sublimation. Nanoparticles in the suspension are preserved during the freezing process through the addition of a cryoprotectant, usually an inert species that sterically prevents particle–particle interactions, overlap, and aggregation.

The lyophilization protocol used herein was the one optimized in our previous study [11]. In brief, HPMC E3 was added to nanoparticle suspensions following FNP at a 1:1 HPMC E3:solids ratio. The E3 acted as a cryoprotectant as the nanoparticle suspension was immersed in a bath of dry ice and acetone (− 78 °C) and rapidly frozen. Frozen samples were then transferred to a − 80 °C freezer overnight. Lyophilization took place in a VirTis AdVantage Pro BenchTop Freeze Dryer (SP Scientific, Pennsylvania, USA) at − 20 °C under vacuum.

### Spray drying conditions

Spray drying was performed using a similar protocol to the one described in Feng et al. [16]. In brief, following nanoparticle formation, HPMC E3 was added to the nanoparticle suspension at a 1:1 HPMC E3:mass ratio to prevent particle aggregation during the drying process. Next, the suspension was fed into a Büchi B-290 spray drier (Büchi Corp., Delaware, USA) via a peristaltic pump at a flow rate of 8 mL/min. Drying parameters such as inlet temperature, mass ratio of added HPMC E3, and aspirator gas flow rate were optimized. The optimal inlet temperature was found to be 145 °C. Following drying, powders were collected and weighed in order to calculate the yield efficiency (YE) of the process. The powder particle size was observed using an Eclipse E200 bright-field microscope (Nikon Instruments, Japan).

### Powder characterization: X-ray powder diffraction (XRPD), differential scanning calorimetry (DSC), and water content

*XRPD:* A D8 Advance diffractometer (Bruker Corporation, Massachusetts, USA) with Ag Kα radiation (λ = 0.56 Å) and a LynxEye-Xe detector was used for XRPD. A polyimide capillary tube (inner diameter = 1 mm) was loaded with 5–10 mg of powder and sealed with quick-setting epoxy. Scattering data were collected over values of 2θ from 3 to 20°, which correspond to Cu Kα 2θ values from 8.2 to 57.0°. A step size of 0.025° (0.067° for Cu Kα radiation) and a rate of 5 s/step were used. Note that in the following sections, all the XRPD results are presented in momentum transfer *Q*, where *Q* is a function of wavelength λ and diffraction angle θ $\left( {Q = \frac{4 \cdot \pi \cdot \sin \left( \theta \right)}{\lambda }} \right)$.

*DSC* A Q200 DSC (TA Instruments, Delaware, USA) was used for DSC measurements. 5–10 mg of sample was weighed into a pan and equilibrated at 20 °C under dry N_2_ atmosphere (50 mL/min). The samples were then heated at 5 °C/min from 20 to 300 °C. The scan was analyzed by TA Instruments Universal Analysis 2000 software.

*Water content* A V20S Compact Volumetric KF Titrator (Mettler Toledo, Ohio, USA) was used to measure the water content of spray dried powders. 20–30 mg of powder was weighed and then deposited into the device’s titration chamber. After 5 min of stirring, the automatic titration process was performed. Aquastar Titrant 5 and Aquastar Combimethanol (EMD Millipore, Massachusetts, USA) were used as titrants with two-component reagents and solvent, respectively.

### OZ439 dissolution

The in vitro solubilization of OZ439 from nanoparticle powders over time in simulated biorelevant media was measured for comparison against unencapsulated OZ439 mesylate. The solubilization protocol was designed to mimic the intended conditions of oral pediatric administration in the developing world; namely, that a mother would add water to the nanoparticle powder before feeding the suspension to an infant.

**Fig. 3:**
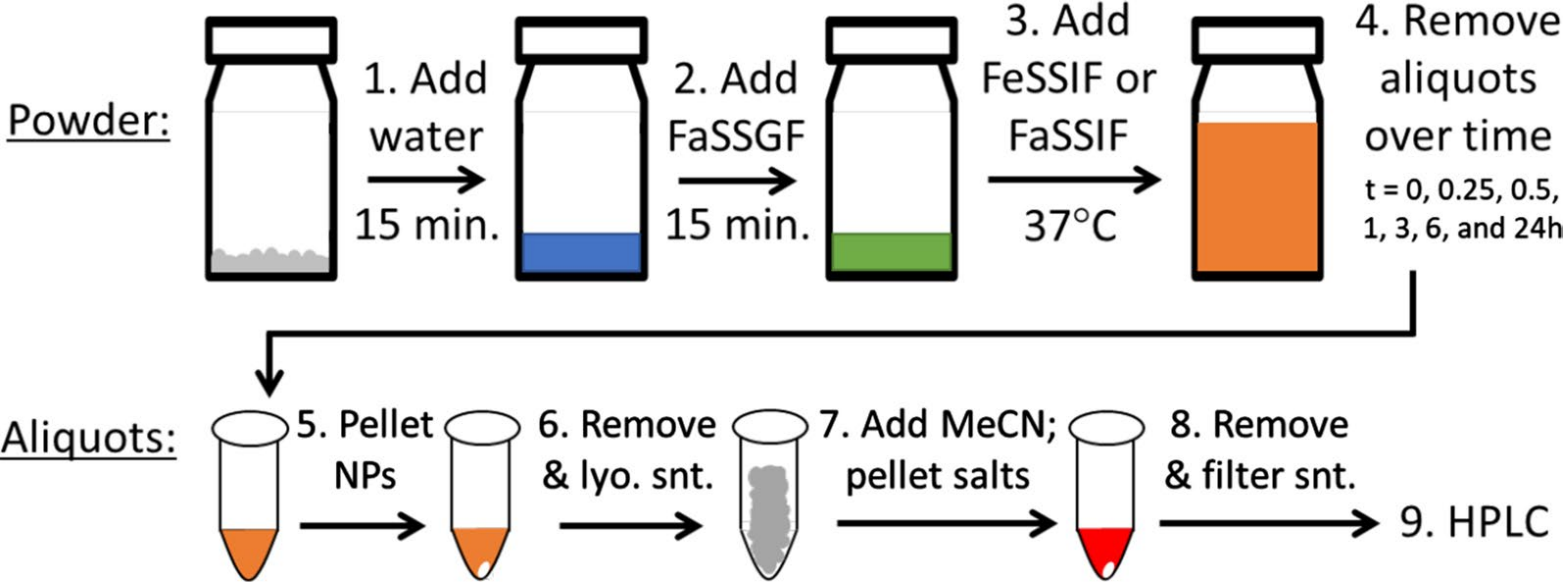
Flow diagram showing steps taken during OZ439 in vitro solubilization tests. Following intestinal fluid addition in step 3, the maximum theoretical concentration of OZ439 was approximately 0.535 mg/mL. Pelleted NPs (after step 5) or bile salts (after step 7) are denoted by white ovals. As dissolution matching 100% of theoretical dissolution was achieved via this protocol, we found that the method results in negligible OZ439 losses despite its several steps

25 mg of powder, containing 3.37 mg OZ439, was weighed into a scintillation vial. 0.515 mL of water was added, and the powder was allowed to redisperse for 15 min (Step 1, Fig. 3). 0.057 mL of concentrated simulated gastric fluid (FaSSGF) was then added, such that the resulting mixture was at the proper pH and salt concentration of gastric fluid, and the suspension was placed in a water bath at 37 °C (Step 2, Fig. 3). After 15 min, 5.72 mL of either fasted-state (FaSSIF) or fed-state (FeSSIF) simulated intestinal fluid was added to the suspension (Step 3, Fig. 3). Thus the total amount of fluid added was 6.29 mL, and the maximum concentration of solubilized OZ439 was approximately 0.535 mg/mL. It should be noted that during long-term stability studies, the maximum possible concentration of OZ439 in a 25 mg powder sample was lowered slightly due to the sample having absorbed water over time; this was accounted for when calculating percent solubilization of OZ439.

After intestinal fluid was added, the suspension remained in a water bath at 37 °C, and 0.8 mL aliquots were removed at t = 0, 0.25. 0.5, 1, 3, 6, and 24 h (Step 4, Fig. 3). Aliquots, which contained bile salts, dissolved OZ439, and nanoparticles, were centrifuged in an Eppendorf Centrifuge 5430R at 28,000 rpm for 10 min to pellet nanoparticles (Step 5, Fig. 3). The supernatant was then removed, frozen, and lyophilized (Step 6, Fig. 3). The lyophilized powder was resuspended in a mixture of acetonitrile and THF (90/10, v/v), which dissolved any OZ439 present, but not residual bile salts. This suspension was sonicated to help dissolve OZ439, then centrifuged to pellet the insoluble bile salts (Step 7, Fig. 3). The supernatant was removed and filtered through a GE Healthcare Life Sciences Whatman™ 0.1 µm syringe filter. OZ439 concentration was determined by high performance liquid chromatography (HPLC) using a Gemini C18 column (particle size 5 μm, pore size 110 Å). The OZ439 detection method used an isocratic mobile phase of 99.95%/0.05% acetonitrile/trifluoroacetic acid at 45 °C and a detection wavelength of 221 nm. OZ439 concentration was calculated from a standard curve. Measurements were performed in triplicate.

Figure 3 shows a flow diagram of the in vitro dissolution test conditions and subsequent OZ439 separation train. The loss of OZ439 throughout the steps was minimal; in several instances, an amount of dissolved OZ439 over 98% of the theoretical maximum was observed.

### Long-term powder stability

For a nanoparticle formulation in dry powder form to be effective at combatting malaria in the developing world, it must retain its superior drug solubilization properties through long-term storage in hot, humid conditions. The tests described below were intended to rapidly age the powders in harsh conditions before assessing their physical characteristics and dissolution kinetics. A future study in the formulation’s development will include temperature cycling and use commercially suitable storage containers and conditions that reflect the real world conditions. Here, three phases of experiments were employed to assess powder stability. First, vials containing lyophilized OZ439 NPs were placed uncapped in an oven at 50 °C and 75% relative humidity (RH). After 1 day, and again after 1 week, aliquots of powder were removed and their OZ439 dissolution kinetics were measured using the protocol above.

In the second phase, vials of spray dried OZ439 NPs were placed in the same conditions (uncapped, 50 °C, 75% RH). OZ439 dissolution was measured after 1, 3, 7, 14, 21, and 28 days. At each time point, some powder was removed for quantification by XRPD, DSC, and titration to determine water content. This phase is referred to as the ‘28-day time course.’

In the third phase, referred to as the ‘6-month time course,’ spray dried OZ439 NPs in capped vials (hand tight, without sealant or tape) were placed in an oven at 40 °C and 75% RH. After 3, 7, 14, and 28 days, and 2, 3, and 6 months, a vial was removed, and OZ439 solubilization was tested and XRPD was performed. In addition, at t = 0, 2, and 6 months, water content was determined and DSC was performed.

## Results

### Nanoparticle formation and characterization

Nanoparticles containing OZ439:oleate and stabilized by HPMCAS 126 were formed by FNP in both the CIJ and MIVM mixers. HPMCAS 126, a cellulosic derivative polymer with acetate and succinate groups along its backbone, was chosen as a stabilizer because of its relatively low cost—approximately two orders of magnitude lower—compared to the block copolymers usually used in FNP [17]. We have previously demonstrated that HPMCAS is a suitable stabilizer for FNP [11, 16, 18]. Sodium oleate, OZ439 mesylate, and HPMCAS 126 were dissolved in a mixture of methanol and THF (1:2, v/v) and rapidly mixed with water. During the mixing, in situ hydrophobic ion pairing took place between oleate anions and OZ439 cations, resulting in a hydrophobic OZ439:oleate complex. HPMCAS 126 and the OZ439:oleate complex nucleated and self-assembled into nanoparticles with a narrow size distribution under both mixing geometries.

**Fig. 4:**
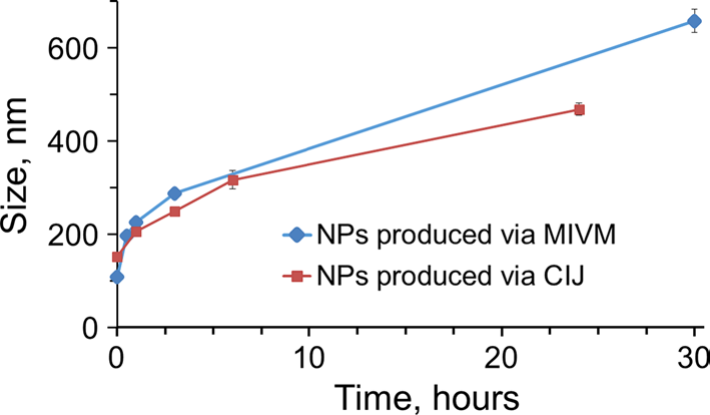
Size over time of nanoparticles produced via FNP either in the CIJ mixer or the MIVM. NPs produced by the CIJ (red squares) were initially larger but ripened more slowly than those produced by the MIVM (blue circles). Nanoparticles produced by both mixers remained in an acceptable size range, i.e. less than 400 nm, and monodisperse 6 h after fabrication and were therefore suitable for additional drying unit operations such as lyophilization or spray drying

In the CIJ, NPs approximately 150 nm in diameter formed (hereafter, ‘CIJ NPs’), and the initial particle size of NPs produced by the MIVM (hereafter, ‘MIVM NPs’) was approximately 100 nm. Over time, NPs produced by both mixers increased in size by Ostwald ripening; the MIVM NPs, which were initially smaller, ripened somewhat more rapidly than the CIJ NPs (Fig. 4). This difference between ripening profiles is consistent with time scale for Ostwald ripening scaling with *R*^3^, which we have demonstrated previously [15]; i.e. smaller particles grow more rapidly.

For our purposes, nanoparticles should remain stable and at the nano-scale for at least 6 h to allow for drying steps such as spray drying or freezing before lyophilizing. Though the HPMCAS-stabilized NPs ripen much more quickly than traditional block copolymer-stabilized NPs produced by FNP, NPs produced by both mixers remained under 400 nm for at least 10 h (Fig. 4). As such, the scaled-up MIVM formulation was deemed acceptable for moving into further processing by spray drying.

### Lyophilization and spray drying

**Fig. 5:**
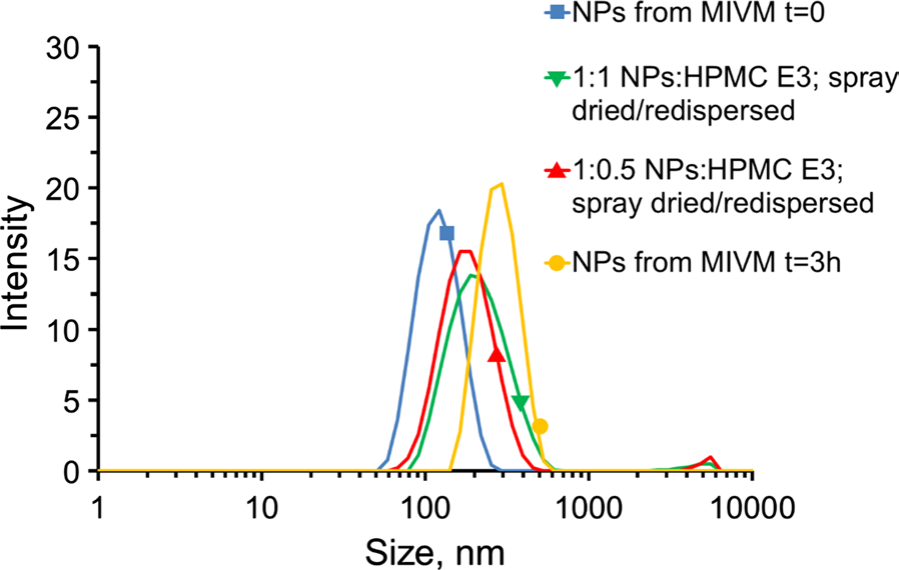
Effect of the amount of HPMC E3 added prior to spray drying on the redispersion of nanoparticles from the spray dried powder. Size distributions of nanoparticles immediately after formation (blue square), 3 h after formation (yellow circle), upon redispersion after spray drying with 0.5 (red triangle) and 1 (green triangle) mass equivalents of added HPMC E3. NPs sprayed 1:1 with HPMC E3 (green) redispersed better then NPs sprayed 1:0.5 with E3 (red), based on the size of the ~ 5000 nm aggregation peak seen by DLS. Both spray dried formulations redispersed to a size smaller than the size to which the original NPs had ripened by 3 h after formation

Lyophilization and spray drying were both optimized to produce a dry powder from the OZ439 NP suspension. In both cases, the addition of HPMC E3 at a 1:1 mass E3:mass solids ratio prior to the drying operation stabilized NPs against aggregation during processing. The size of NPs in suspensions of redispersed lyophilized powder has been shown previously [11]. For spray drying, multiple ratios of E3 were tested: when 0.5 equivalents or 1 equivalent (by mass) of E3 were added, the resulting dry powders redispersed to NPs in water. In both cases, the redispersed NPs were smaller on average than the size to which fresh NPs from the MIVM had ripened by three hours (Fig. 5). Ideally, the outlet from an MIVM will be fed directly into a spray drier to minimize the effect of size growth. However, at the lab scale the liquid flow rates from the CIJ or MIVM are greater than the drying rates that can be achieved by the lab-scale spray drier. Thus, in these tests, the MIVM was run in a batch mode, producing 350 mL of NP suspension in a batch in 2.5 min. This batch was then spray dried over 40 min, during which some ripening took place. It is, therefore, imprecise to compare the size of reconstituted NPs with the original output of the MIVM, which is why we note that the reconstituted NPs fall within an acceptable and expected size range.

Once spray drying parameters had been optimized, a large volume of NP suspension (~ 1500 mL) was dried in preparation for the long-term stability studies. The yield efficiency of this process, calculated by the equation below, was 45 ± 5%. This is expected to increase with batch size in a full-scale process.

$${\text{Yield efficiency }}\left( {\text{\% }} \right) = \frac{{{\text{mass}}\;{\text{of collected spray dried powder}}}}{\text{mass of solids fed to spray drier}} \times 100$$

**Fig. 6:**
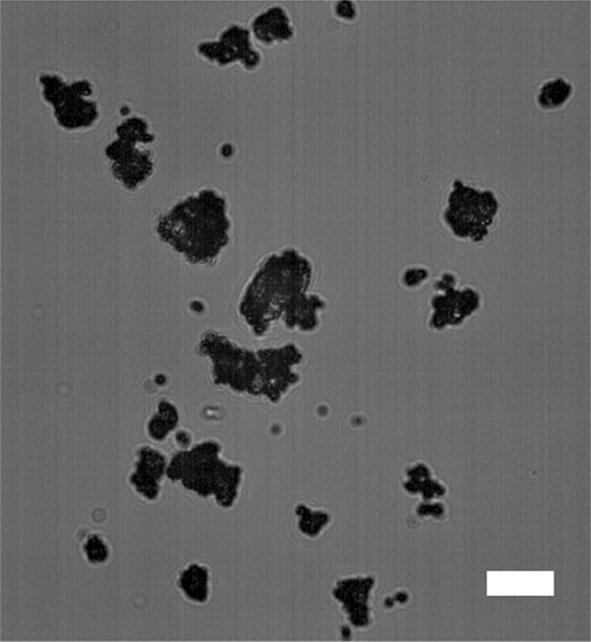
Bright-field microscopy image of the spray-dried HPMCAS NP powders (mass ratio of NP:HPMC E3 = 1:1). The scale bar is 10 µm

As measured by microscopy, spray-drying produced fine particles with median diameter of 7.8 μm based on number distribution. The morphology of the spray-dried powders was observed to be shriveled, instead of dense spheres (Fig. 6). During the fast drying at high temperature, NPs accumulated on the droplet surface and formed a shell, which further buckled due to the capillary force of the shrinking droplet. The wrinkled surface may increase the surface area and hence the wettability, assisting redispersity in water. This morphology observation is also consistent with our previous work [16, 18].

### OZ439 solubilization and dissolution

**Fig. 7:**
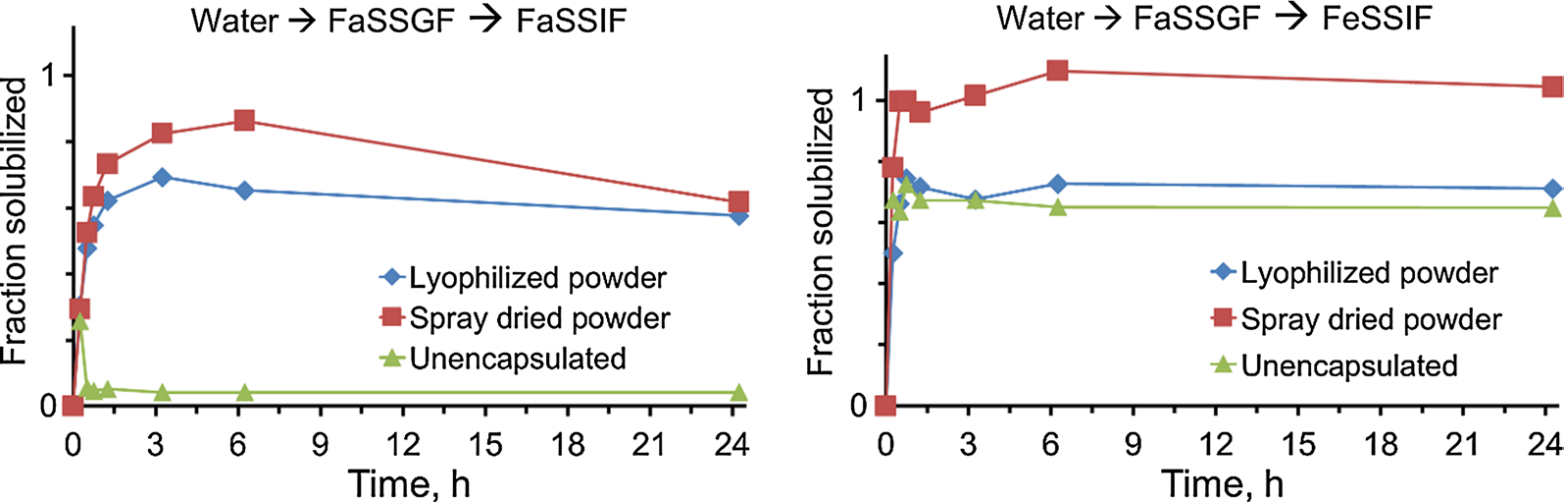
Dissolution kinetics of OZ439 when unencapsulated (green triangles) or encapsulated into nanoparticles via FNP and processed into a dry powder by lyophilization (blue diamonds) or spray drying (red squares). Spray dried NPs achieved up to 20-fold superior OZ439 solubilization compared to OZ439 mesylate powder in FaSSIF, and also outpaced lyophilized NPs by up to 1.3 times

The in vitro dissolution of OZ439 from lyophilized or spray dried nanoparticles in simulated biorelevant media was determined and compared to the OZ439 mesylate powder dissolution under the same conditions. When swapped from water through FaSSGF to FaSSIF, spray dried nanoparticles exhibited dissolution superior to both unencapsulated powder and lyophilized NPs (Fig. 7). Spray dried NPs achieved over 20-fold higher solubilized OZ439 than unencapsulated powder after 6 h and solubilized up to 86% of the OZ439 in the system. Since OZ439’s solubility limit in FaSSIF is approximately 140 µg/mL (0.26 on the y-axis in Fig. 7), both the spray dried and lyophilized NPs achieved OZ439 supersaturation after 1 h and maintained this state for the duration of the study. The decrease in solubilization after 24 h can be explained by possible recrystallization from the supersaturated system.

When swapped from water through FaSSGF into FeSSIF, unencapsulated powder and lyophilized NPs exhibited similar dissolution profiles. Spray dried NPs, in contrast, achieved 100% solubilization by 0.5 h and maintained this state for the duration of the study. OZ439 solubility in FeSSIF is higher than in FaSSIF (2.5 mg/mL vs. 0.14 mg/mL), so the system was not supersaturated and never demonstrated recrystallization.

In both FaSSIF and FeSSIF, spray dried NPs provide more complete OZ439 solubilization than either lyophilized NPs or unencapsulated powder. In so doing, spray dried NPs may be an effective means of minimizing the ‘food effect,’ i.e. the difference in OZ439 solubilization between the fed and fasted states. By reducing this difference, our NPs may remove or reduce the necessity of co-administering OZ439 with enough food to induce fed-state GI conditions. Simplifying administration in this way is particularly important for pediatric malaria patients, who have poor appetites and may have difficulty eating the quantity of food required. Additionally, reducing the food effect should reduce variability in drug PK and efficacy in vivo, since variable GI conditions will have less impact on drug solubilization.

In the case of both FaSSIF and FeSSIF, spray dried NP powders achieved faster and more complete OZ439 solubilization than lyophilized powders. This phenomenon may be due to wettability issues that arose during the course of small-scale lyophilization. At the walls and bottom of the glass vial in which they were dried, lyophilized samples sometimes formed a dense lyophilization cake that was difficult to redisperse. Another possible explanation for the difference in performance between the powders may arise from the HPMCAS’ ability to protect nanoparticles from aggregation during lyophilization. In our previous study, we found that adding HPMC E3 equivalent to 1:1 solids prior to freezing and lyophilizing helped with redispersibility; nevertheless, a small population of aggregates was observed, which may have hindered the powder’s ability to enhance OZ439 solubilization.

The grade of HPMCAS used herein has been optimized for formulating spray dried dispersions and hot melt extrusions, but this alone may not explain the poorer performance of lyophilized powders compared to spray dried powders. Chiang et al. found no significant difference in in vivo performance between dried HPMCAS-based dispersions of Griseofulvin processed by spray drying and lyophilization [19]. In our case, nanoparticle aggregation during freezing or lyophilization has the potential for reducing OZ439 solubilization, as mentioned above; this was not a consideration for Chiang et al., whose formulation did not use nanoparticles.

### Long-term powder stability

**Fig. 8:**
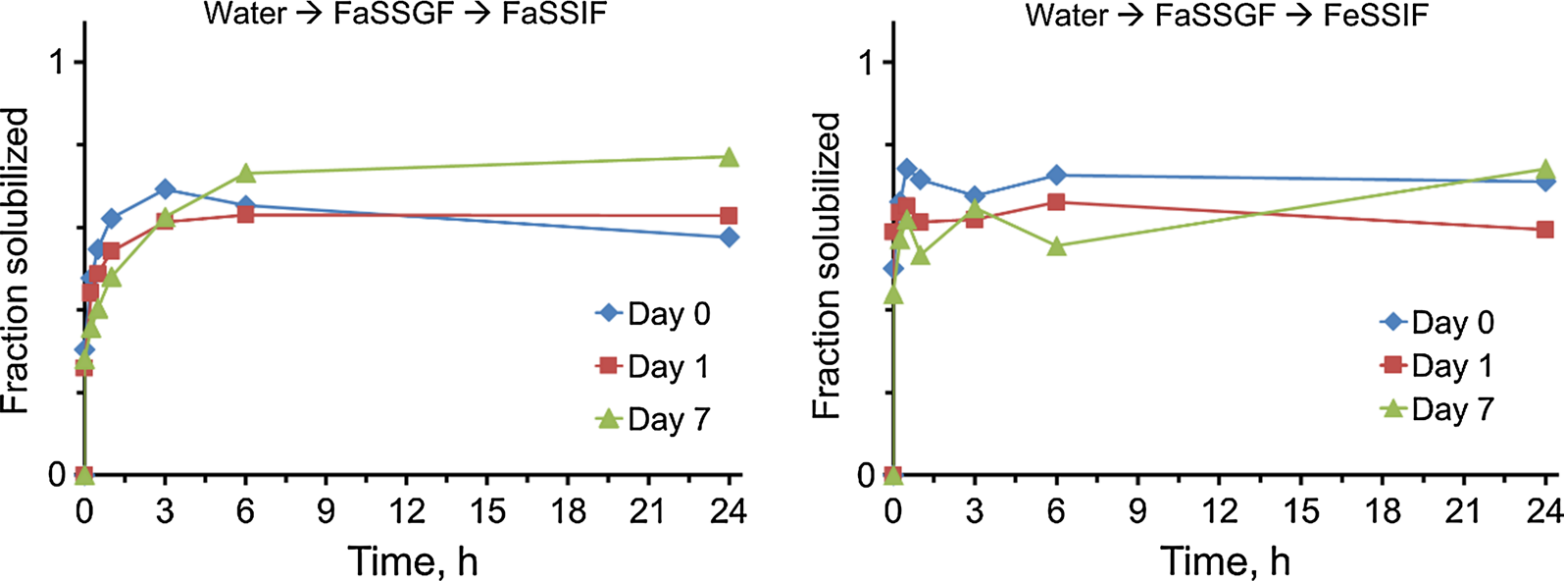
Dissolution kinetics of lyophilized OZ439 NP powder after storage in an oven at 50 °C and 75% RH in uncapped vials. Though the powder’s appearance changed radically after 1 day in the oven (see Additional file 1: Figure S1), the dissolution kinetics of encapsulated OZ439 remained largely the same over the course of a week in these conditions. After 1 day (red squares) and 7 days (green triangles) in the oven, OZ439 dissolution kinetic profiles matched those of the powder immediately after lyophilization, both in terms of completeness and shape. In all cases, 60–70% of OZ439 was solubilized, with NPs FeSSIF reaching this plateau faster than NPs in FaSSIF

Lyophilized NPs powders were placed in an oven at 50 °C and 75% RH in uncapped vials for up to 1 week. The in vitro solubilization of OZ439 was assessed on the powder prior to, after 1 day in, and after 7 days in the oven. OZ439 dissolution remained constant across this period, despite the potential for water uptake by the HPMCAS stabilizer in the powders (Fig. 8). Unlike hot melt extrusions, in which drug fused to the HPMCAS backbone would, upon the hydration of that backbone, potentially diffuse throughout the polymer matrix and crystallize, in our nanoparticle system we expect discrete regions of drug to be distributed throughout the HPMCAS matrix from the onset. Thus, the drug does not gain freedom to diffuse upon HPMCAS hydration, and remains in its initial state despite water uptake.

**Fig. 9:**
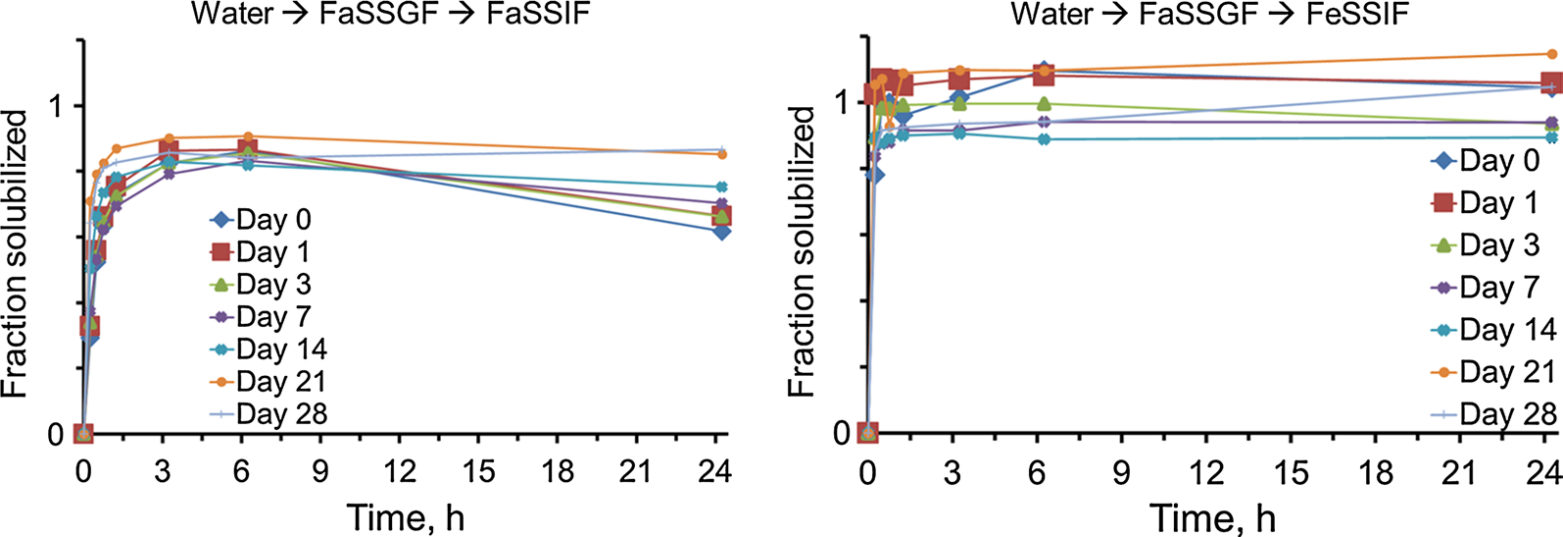
Dissolution kinetics of spray dried OZ439 NP powder after storage in an oven at 50 °C and 75% RH in uncapped vials. In all cases, NPs in FaSSIF achieved 80–90% maximum OZ439 solubilization, and NPs in FeSSIF reached 90–100% solubilization. Though there is more variability in the FeSSIF results (right), no trend of decreasing activity as a function of incubation time is observed

Spray dried powders, when subjected to these same oven conditions over the course of a month, also retained their OZ439 dissolution profiles (Fig. 9). After 1, 3, 7, 14, 21, and 28 days, aliquots were removed from the oven for in vitro solubilization tests and XRPD. There was no discernable trend toward loss of solubilization as a function of time in the oven, and solubilization profiles after 28 days in these harsh conditions are largely the same as before the test began.

**Fig. 10:**
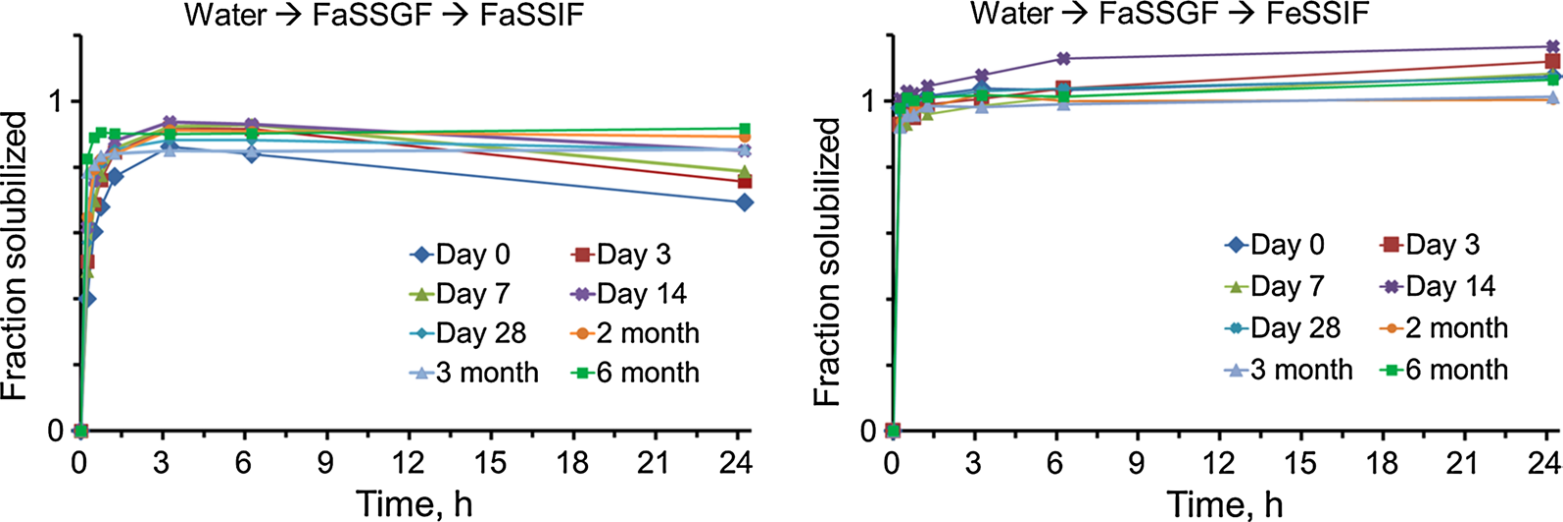
Dissolution kinetics of spray dried OZ439 NP powder after storage in an oven at 40 °C and 75% RH in capped vials. In all cases, NPs in FaSSIF achieved 80–90% maximum OZ439 solubilization, and NPs in FeSSIF achieved complete solubilization

Through the 6-month time course at 40 °C and 75% RH, the spray dried nanoparticle powder retained its in vitro OZ439 solubilization potential (Fig. 10). As in the 1-month course, OZ439 solubilization at the end of the time course is the same as before the powder was exposed to the oven. It should be noted that the dissolution kinetics did not change despite some water uptake by the powder over time (Table 1).

**Table 1:** Water uptake by spray dried NP powder over 6-month stability time course

Time in oven	0 days	2 months	6 months
Water content (w/w)	Below detection limit (< ~ 4%)	Below detection limit (< ~ 4%)	6.26%

Immediately following spray drying and after 2 months in the oven, water content was below the detection limit of the KF titrator. By the end of 6 months, the powder had accumulated a small amount of water, which did not adversely affect OZ439 solubilization

**Fig. 11:**
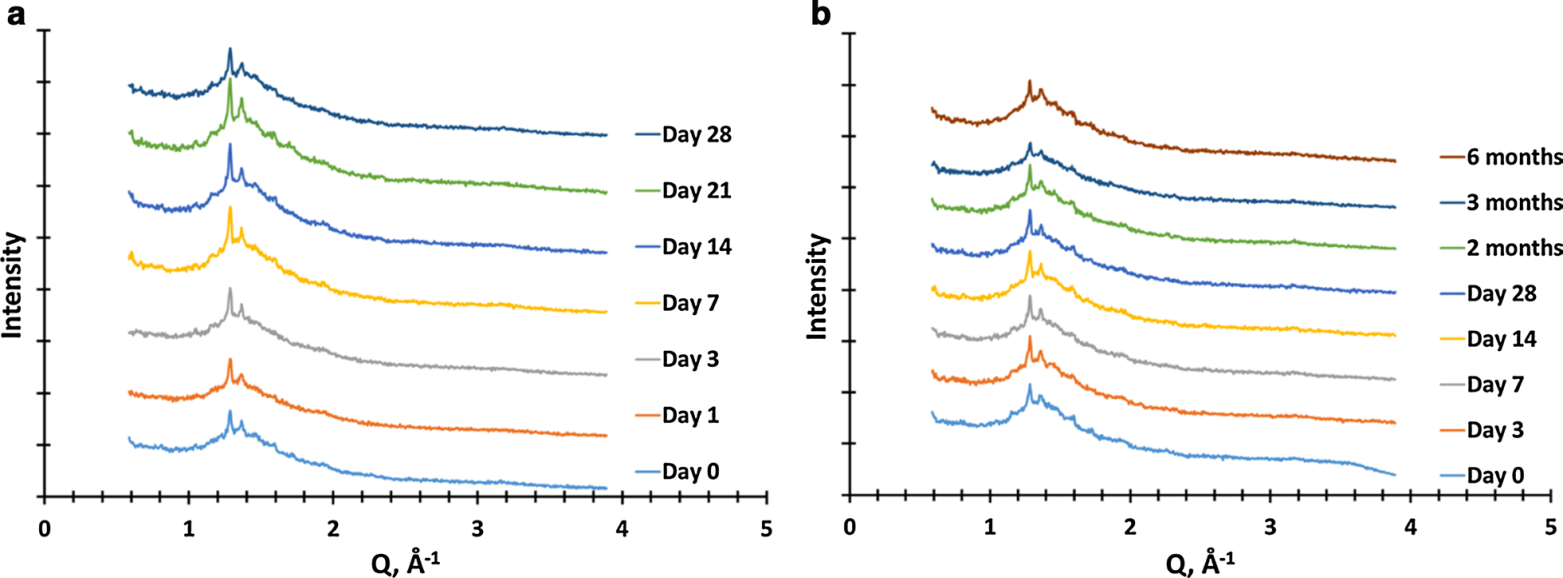
XRPD of spray dried OZ439 NP powder after oven storage at **a** 50 °C and 75% RH in uncapped vials for a month and **b** 40 °C and 75% RH in capped vials for 6 months. Distinct Bragg peaks are observed, but do not change in intensity or width over time. Individual profiles are offset vertically to facilitate comparison

XRPD results from each time throughout the (a) 28-day and (b) 6-month time courses are reported in Fig. 11. The samples are shown to contain some degree of crystallinity, indicated by sharp Bragg peaks at Q = 1.3 and 1.4 Å^−1^. Importantly, neither these peaks nor the overall profiles of the powder across over time appear to change significantly, again demonstrating powder stability. These peaks likely are due to a sodium mesylate salt formed during drying from spectator sodium and mesylate ions. See Additional file 1: Figure S2 for the XRPD profiles of the individual components used in the study, which can be compared to the profiles of the powder at t = 0 and sodium mesylate.

**Fig. 12:**
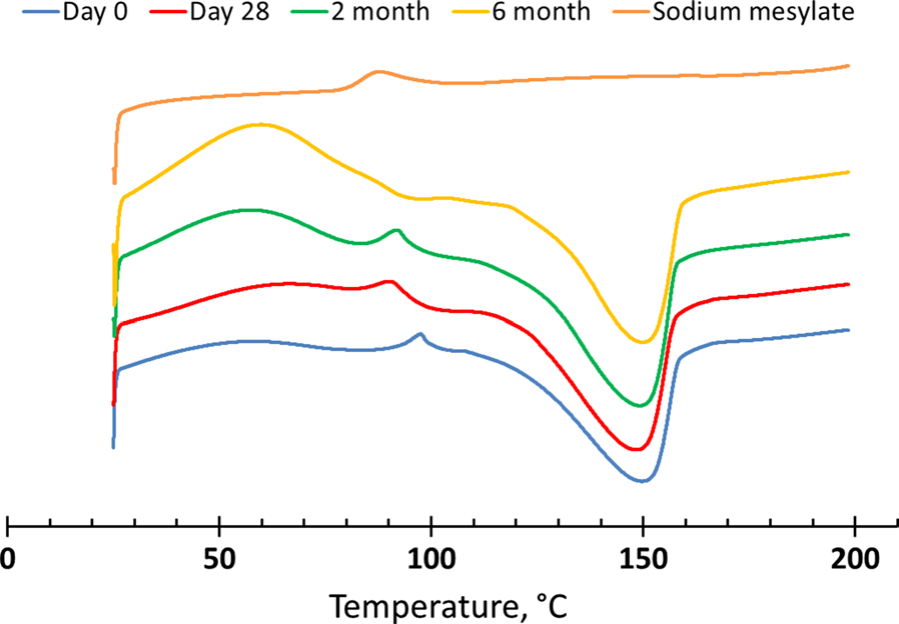
DSC profiles of spray dried OZ439 NP powder after oven storage at 40 °C and 75% RH in capped vials for 6 months. Profiles are similar across 6 months, with the exception of the small peak at 90 °C, which was initially present but disappears by 6 months. This peak corresponds to sodium mesylate, which may be formed from spectator sodium and mesylate ions during drying and disappears over time due to water uptake

DSC results from the 6-month time course are reported in Fig. 12. The profiles closely match one another, with the exception of a peak at 90 °C matching sodium mesylate. This broadens and disappears by 6 months, potentially because of water uptake by hygroscopic sodium mesylate.

## Discussion

The work presented herein demonstrates that the lab-scale nanoparticle formulation of the potent antimalarial OZ439 can be scaled up using industrially relevant unit operations. As before, Flash NanoPrecipitation with hydrophobic ion pairing was used to form nanoparticles stabilized by HPMCAS and containing a hydrophobic complex of OZ439 and oleate. The limitation of the dilution step following nanoparticle formation in a two-stream confined impinging jet mixer was overcome by forming NPs in an industrial-scale four stream multi-inlet vortex mixer, which was operated at 160 mL/min and can be operated at up to 1.5 L/min. The lyophilization drying unit operation used previously was replaced with scalable spray drying, which formed nanoparticle powders that redispersed to nano-scale in water and showed in vitro OZ439 solubilization superior to that of both un-encapsulated OZ439 mesylate and lyophilized nanoparticle powders. The spray dried powder also demonstrated robust stability, maintaining its XRPD, DSC, and solubilization profiles over 28 days in harsh conditions (50 °C, 75% RH, uncapped) and for 6 months in accelerated conditions (40 °C, 75% RH, capped).

Considering the scale of malaria therapeutics produced worldwide each year, to be industrially relevant, any process to formulate OZ439 must be scalable to at least the scale of hundreds or thousands of kilograms of drug product per year. The steps taken here are a move toward a fully scalable process. FNP and spray drying are both continuous unit operations, which will aid significantly in future efforts to scale the process up. We have demonstrated scalability of our multi-inlet vortex mixer to operate at flow rates of more than 5 L/min, and even larger units can be readily designed through simple geometric and flow rate scale-up. The next steps for scaling up this particular formulation are to go to the pilot scale for GMP production of powders that can be evaluated for in vivo exposure in humans.

Another major consideration for a scalable process is the cost of goods. This FNP formulation effectively adds three excipients to OZ439—sodium oleate, HPMCAS-126, and HPMC E3—all of which add minimal cost to the final product. These excipients and their grades were chosen specifically because of their low costs; all three are available at scale for $10–100 per kilogram. Moreover, it should be noted that the potential benefits of a single-dose cure for malaria may justify slightly higher production costs for a therapy than traditional multi-dose regimens due to improved compliance. The acceptable range for cost of goods was published in the TPP paper published in 2017 [20].

The aging studies included herein are not intended to precisely mimic environmental conditions in endemic countries where this formulation would eventually be used, but are instead intended to quickly age the formulation in a consistently harsh environment. Stability tests reflective of actual environmental conditions would include temperature cycling studies in commercially suitable containers. These tests are planned for a later part of this formulation’s development.

It should be noted that in vitro dissolution kinetics using biorelevant media, as performed here, are the most accurate way to predict in vivo drug absorption in humans. OZ439 has a unique PK profile, with low oral bioavailability in humans, but significantly high oral bioavailability in all animal models tested to date (greater than 80%, regardless of formulation). Therefore, to obtain useful in vivo data, a formulation must be tested in humans, requiring GMP manufacturing. These experiments are part of the future plans for this formulation, and were beyond the scope of this paper, which focused on formulation, scale-up, and physical stability.

The formulation and method development in this study may offer an inexpensive and scalable means of improving the oral bioavailability of OZ439 and help the drug realize its potential as a single-dose oral malaria therapeutic. Future work will include an investigation of concentrating the nanoparticle suspension following its formation in the MIVM and prior to its entry into the spray dryer. Pre-concentrating the NP dispersion would reduce spray drying requirements in terms of time and cost. To this end, we will next investigate the impact of continuous tangential flow ultrafiltration (TFF) on the stability of the NP formulation. Additional unit operations such as flash evaporation, which will reduce the volume of organic solvent in the NP suspension and further stabilize NPs from Ostwald ripening, may be required in conjunction with TFF.

## Additional file

**Additional file 1: Figure S1.** Lyophilized NP powder (left) before being placed in an oven uncapped at 50 °C and 75% RH and (right) after 1 day in the oven. **Figure S2.** XRPD profiles of the raw individual components used in the study, along with the t = 0 spray dried nanoparticle powder (light blue, bottom). The signal of ‘OZ439 oleate, etc.’ was obtained by physically mixing OZ439 mesylate dissolved in methanol and sodium oleate dissolved in methanol with water. The resulting solution became cloudy, indicating the formation of an insoluble OZ439:oleate complex. The solution was dried and XRPD was performed. This profile can be thought of as a physical mixture of sodium oleate, sodium mesylate, OZ439 mesylate, and OZ439 oleate. The peaks at Q = 1.3, 1.4, and 1.6 nm^−1^ in the NP powder align closely with similar peaks in sodium mesylate (green, second from top), suggesting these peaks are due to sodium mesylate that formed from spectator sodium and mesylate ions during drying. These sodium mesylate crystals likely formed outside the NPs and are not associated with the amorphous OZ439:oleate core.

## Abbreviations

NPs: nanoparticles
MMV: Medicines for Malaria Venture
BMGF: Bill and Melinda Gates Foundation
API: active pharmaceutical ingredient
HIP: hydrophobic ion pairing
FNP: Flash NanoPrecipitation
HPMCAS: hydroxypropyl methylcellulose acetate succinate
HPLC: high performance liquid chromatography
FaSSGF: fasted-state simulated gastric fluid
FaSSIF: fasted-state simulated intestinal fluid
FeSSIF: fed-state simulated intestinal fluid
CIJ: confined impinging jets
MIVM: multi-inlet vortex mixer
DI: deionized water
XRPD: x-ray powder diffraction
DSC: differential scanning calorimetry
RH: relative humidity
GI: gastrointestinal
PK: pharmacokinetics
