# Supplementary material for: Spray drying OZ439 nanoparticles to form stable, water-dispersible powders for oral malaria therapy
Source: J Transl Med. 2019 Mar 22;17:97. doi: 10.1186/s12967-019-1849-8 (PMC6431012; doi:10.1186/s12967-019-1849-8)
Supplement: Supplementary file 1 — Additional file 1: Figure S1. Lyophilized NP powder (left) before being placed in an oven uncapped at 50 °C and 75% RH and (right) after 1 day in the oven. Figure S2. XRPD profiles of the raw individual components used in the study, along with the t = 0 spray dried nanoparticle powder (light blue, bottom). The signal of ‘OZ439 oleate, etc.’ was obtained by physically mixing OZ439 mesylate dissolved in methanol and sodium oleate dissolved in methanol with water. The resulting solution became cloudy, indicating the formation of an insoluble OZ439:oleate complex. The solution was dried and XRPD was performed. This profile can be thought of as a physical mixture of sodium oleate, sodium mesylate, OZ439 mesylate, and OZ439 oleate. The peaks at Q = 1.3, 1.4, and 1.6 nm−1 in the NP powder align closely with similar peaks in sodium mesylate (green, second from top), suggesting these peaks are due to sodium mesylate that formed from spectator sodium and mesylate ions during drying. These sodium mesylate crystals likely formed outside the NPs and are not associated with the amorphous OZ439:oleate core. [file 12967_2019_1849_MOESM1_ESM.docx]

Supporting information for

Spray drying OZ439 nanoparticles to form stable,
water-dispersible powders for oral malaria therapy

Kurt D. Ristroph^1^, Jie Feng^1^, Simon A. McManus^1^, Yingyue Zhang^1^, Kai Gong^2,3^, Hanu Ramachandruni^4^, Claire E. White^2,3^, Robert K. Prud’homme^1^

*^1^Department of Chemical and Biological Engineering, ^2^Department of Civil and Environmental Engineering, ^3^Andlinger Center for Energy and the Environment, Princeton University, Princeton, New Jersey 08854, United States.*

*^4^Medicines for Malaria Venture, Route de Pré-Bois 20, 1215 Meyrin, Switzerland*

Author e-mails, respectively: [ristroph@princeton.edu](mailto:ristroph@princeton.edu), [jiefeng@princeton.edu](mailto:jiefeng@princeton.edu), [smcmanus@princeton.edu](mailto:smcmanus@princeton.edu), [yingyuez@princeton.edu](mailto:yingyuez@princeton.edu), [kaig@princeton.edu](mailto:kaig@princeton.edu), [ramachandrunih@mmv.org](mailto:ramachandrunih@mmv.org), [whitece@princeton.edu](mailto:whitece@princeton.edu), [prudhomm@princeton.edu](mailto:prudhomm@princeton.edu)


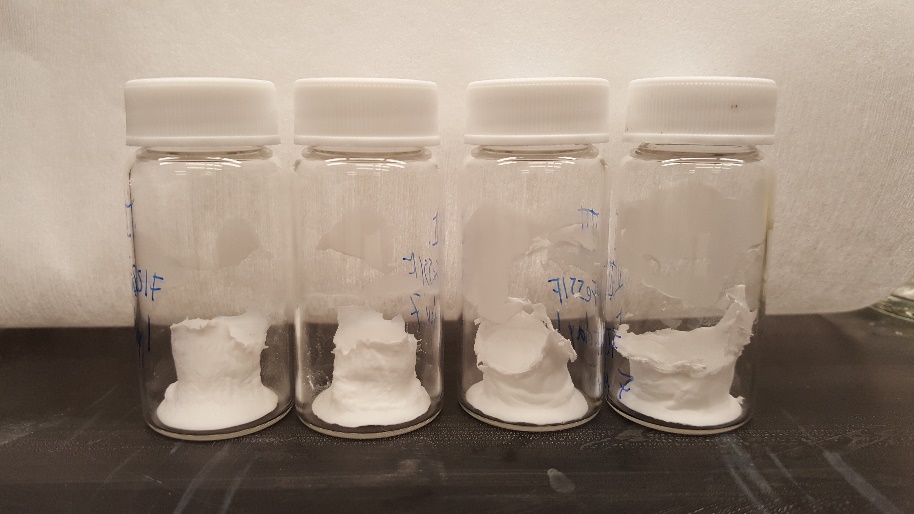

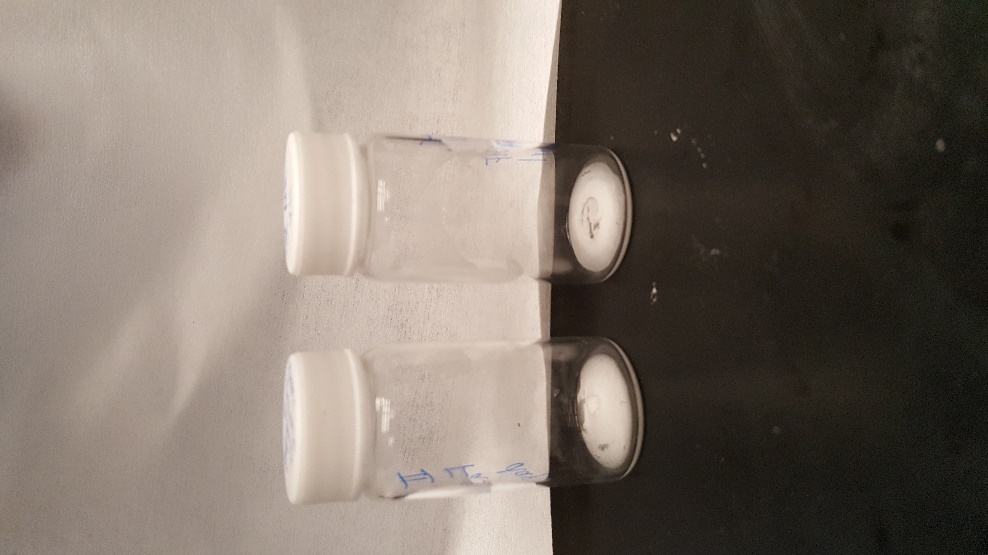


**Figure S1.** Lyophilized NP powder (left) before being placed in an oven uncapped at 50°C and 75% RH and (right) after 1 day in the oven.

**
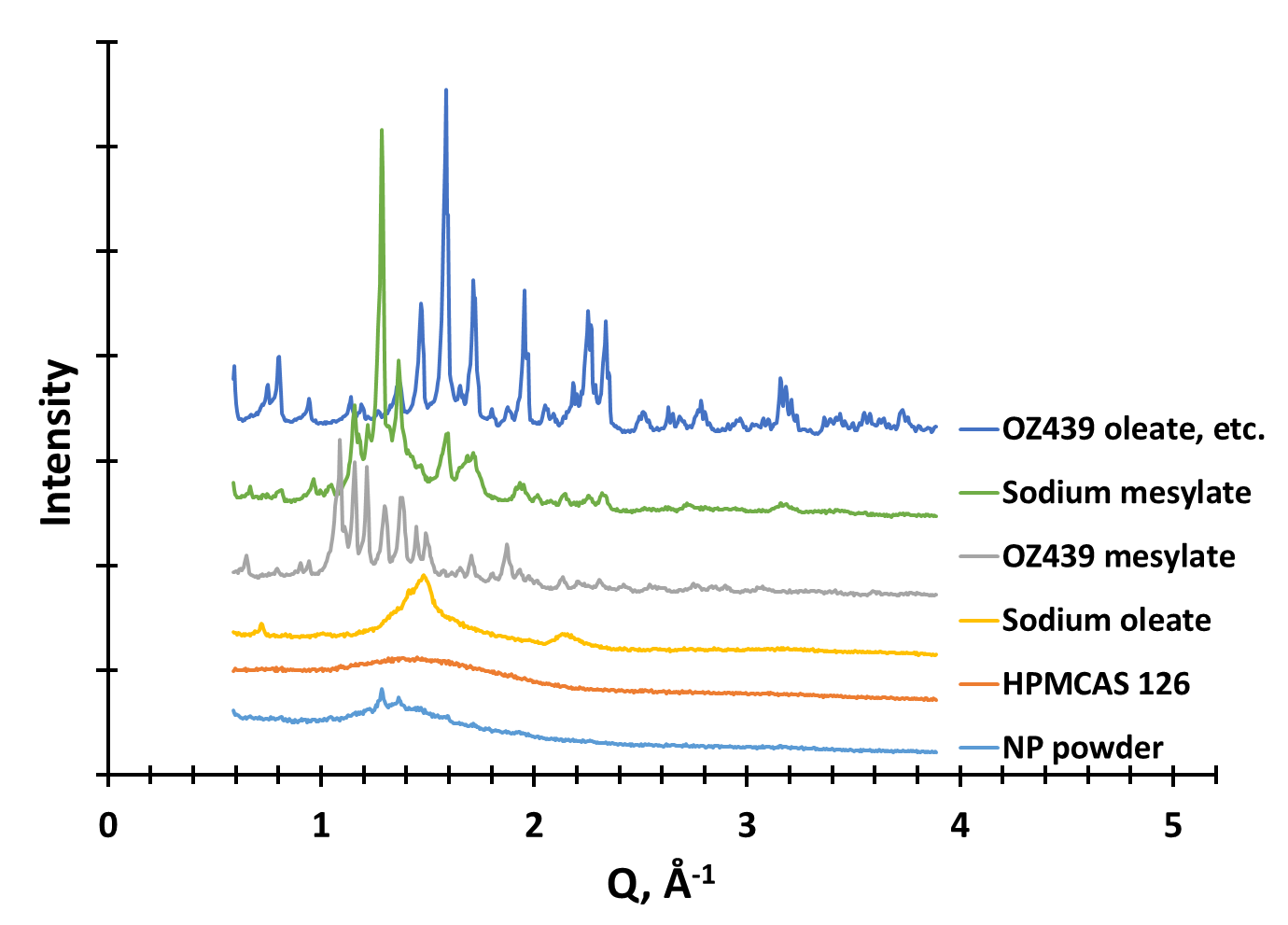
**

**Figure S2.** XRPD profiles of the raw individual components used in the study, along with the t=0 spray dried nanoparticle powder (light blue, bottom). The signal of ‘OZ439 oleate, etc.’ was obtained by physically mixing OZ439 mesylate dissolved in methanol and sodium oleate dissolved in methanol with water. The resulting solution became cloudy, indicating the formation of an insoluble OZ439:oleate complex. The solution was dried and XRPD was performed. This profile can be thought of as a physical mixture of sodium oleate, sodium mesylate, OZ439 mesylate, and OZ439 oleate. The peaks at Q = 1.3, 1.4, and 1.6 nm^-1^ in the NP powder align closely with similar peaks in sodium mesylate (green, second from top), suggesting these peaks are due to sodium mesylate that formed from spectator sodium and mesylate ions during drying. These sodium mesylate crystals likely formed outside the NPs and are not associated with the amorphous OZ439 oleate core.
